# FN-Identify: Novel Restriction Enzymes-Based Method for Bacterial Identification in Absence of Genome Sequencing

**DOI:** 10.1155/2015/303605

**Published:** 2015-12-31

**Authors:** Mohamed Awad, Osama Ouda, Ali El-Refy, Fawzy A. El-Feky, Kareem A. Mosa, Mohamed Helmy

**Affiliations:** ^1^Department of Biotechnology, Faculty of Agriculture, Al-Azhar University, Cairo 11651, Egypt; ^2^Department of Information Technology, Faculty of Computer and Information Sciences, Mansoura University, Mansoura 35516, Egypt; ^3^Department of Applied Biology, College of Sciences, University of Sharjah, P.O. Box 27272, Sharjah, UAE; ^4^Donnelly Centre for Cellular and Biomedical Research, University of Toronto, Toronto, ON, Canada M5S 3E1

## Abstract

Sequencing and restriction analysis of genes like 16S rRNA and HSP60 are intensively used for molecular identification in the microbial communities. With aid of the rapid progress in bioinformatics, genome sequencing became the method of choice for bacterial identification. However, the genome sequencing technology is still out of reach in the developing countries. In this paper, we propose FN-Identify, a sequencing-free method for bacterial identification. FN-Identify exploits the gene sequences data available in GenBank and other databases and the two algorithms that we developed, CreateScheme and GeneIdentify, to create a restriction enzyme-based identification scheme. FN-Identify was tested using three different and diverse bacterial populations (members of* Lactobacillus*,* Pseudomonas*, and* Mycobacterium* groups) in an* in silico* analysis using restriction enzymes and sequences of 16S rRNA gene. The analysis of the restriction maps of the members of three groups using the fragment numbers information only or along with fragments sizes successfully identified all of the members of the three groups using a minimum of four and maximum of eight restriction enzymes. Our results demonstrate the utility and accuracy of FN-Identify method and its two algorithms as an alternative method that uses the standard microbiology laboratories techniques when the genome sequencing is not available.

## 1. Introduction

Bacterial identification is an important routine in the clinical and industrial microbiology laboratories. Microbiologists and researchers stepped up their efforts to improve and facilitate the rapid characterization of various microbial communities. Traditional bacterial identification strategies are mainly based on morphological, biochemical, enzymatic, antigenic, staining, and antibiogram characterization [1]. However, these strategies are time consuming and sometimes fail to identify the bacteria accurately [2]. Many other strategies appear to have improved bacterial identification accuracy, such as automated cellular fatty acid (CFA) analysis, yet these strategies require expensive system and standardized culture condition. Moreover, it cannot differentiate closely related species such as* Escherichia coli* and* Shigella* [2]. Protein analysis and phage analysis are also used as methods for bacterial identification [3]. With the presentation and rapid progress of molecular biology and molecular markers, several new and enhanced bacterial identification methods were developed. These methods include plasmid analysis [4], restriction fragment length polymorphism (RFLP) [5], pulse-field gel electrophoresis (PFGL) [6], random amplified polymorphism DNA (RAPD) [7], fluorescent* in situ* hybridization (FISH) [8], and DNA Props [9].

In the early 1980s, polymerase chain reaction (PCR) provided novel approaches for bacterial identification through amplification of specific sequences/genes from the bacterial genome. Several ribosomal RNA (rRNA) genes and Internal Transcribed Spacers (ITSs) had been utilized for PCR-based bacterial identification such as 16S rRNA, 23S rRNA, 5S rRNA, and SSU rRNA [8, 10]. The PCR-based identification uses the ribosomal genes, since ribosomal genes play an important role in living organisms and have functional stability over evolution ages due to rare variation in its sequences through millions of years, which makes them suitable to be used for identification and taxonomical purposes.

Numerous ribosomal RNA genes and ITSs such as Hsp65, rpoB, gyrB, groEL, and recA have been tested as a genetic marker in bacterial identification [11]. However, 16S rRNA is the most widely used ribosomal RNA genes in bacterial identification due to several reasons: (1) the 16S rRNA gene presents in almost all bacterial families; (2) it has functional and evaluation stability; (3) in many cases, multiple copies of the 16S rRNA gene presented in the genome and sometimes differences in sequences present as well, which can be used to distinguish closely related species; (4) the sequence length is about 1500 : 1550 bp, which is enough for taxonomical purpose and suitable for amplification; (5) the 16S rRNA gene sequence contains conserved regions and variable regions; therefore, it is possible to design a universal primer on these conserved regions for gene sequence amplification [1, 12]. Therefore, several methods for 16S rRNA amplification and analysis were developed: ribotyping [13], denaturing gradient gel electrophoresis (DGGE) [14, 15], temperature gradient gel electrophoresis (TGGE) [15], amplified ribosomal DNA restriction analysis (ARDRA) [16], and terminal restriction fragment length polymorphism (T-RFLP) [17, 18].

With the rapid progress in DNA and RNA sequencing technology, sequencing of 16S rRNA gene and several other genes became a popular method for bacterial identification and phylogenetic reconstruction. Furthermore, it is employed in nucleic acid-based detection, quantification of microbial diversity, and discovery of novel bacterial isolates in different microbiology laboratories [19–22].

Despite the outstanding advancements in speed and accuracy and the remarkable decrease in cost of the sequencing technologies in the recent years, sequencing technologies in developing countries are out of reach for the majority of clinical and research laboratories. This is mainly due to the high cost of establishing sequencing facility and high cost of reagents and maintenance [23–25]. Furthermore, the lack of trained personnel and the limited access to up-to-date scientific information play an important role in constraining the use of such indispensable technology in many clinical and industrial microbiology laboratories in these countries [26, 27]. Most labs depend on outsourcing the DNA/RNA sequencing through using commercial services. Typically, the sample is prepared and sent to a local company that sends it to companies in the EU or China to be sequenced and the results are sent back. Based on our observations, this process is expensive and time consuming (up to several months) and can fail at any point.

**Figure 1:**
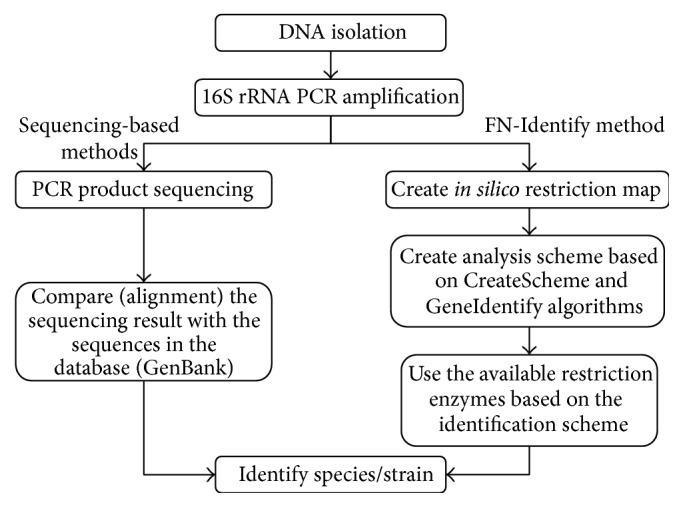
Comparison between sequencing-based identification approach and FN-Identify proposed approach.

In this work, we present a FN-Identify, an efficient and sequencing-free bacterial identification method, as a proposed alternative that can be employed when genome sequencing is inaccessible. FN-Identify, which stands for fragment number-identify, is based on techniques that are available in most of the standard microbiological laboratories. Our new method depends on sequences available in GenBank and other public databases, such as RDP-II [28], Silva [29], and Greengenes [30], restriction enzymes, and the two FN-Identify algorithms that we developed (Figure 1). We used bacterial population of 33 members (species and strains) of* Lactobacillus* genus to develop the method and used two other bacterial populations of 33 and 22 members (species and strains) of* Pseudomonas* and* Mycobacterium*, respectively, to test the method. FN-Identify successfully identified and differentiated all the species/strains using two different genes 16S rRNA and HSP60, in two independent analyses. The identification scheme and the utilized restriction enzymes, created by FN-Identify, demonstrate its efficiency as a rapid, accurate, and affordable alternative method for bacterial identification in the absence of the sequencing technologies.

## 2. Materials and Methods

### 2.1. Bacterial Genomes

We downloaded the 33, 33, and 22* Lactobacillus*,* Pseudomonas*, and* Mycobacterium* members, respectively, with full genome sequences and annotations from Genome Database of the National Center for Biotechnology Information (NCBI) (September 2013).  Table 1 shows the names and GenBank accession number of the* Lactobacillus* members and Tables S1 and S3 (see Supplementary Material available online at http://dx.doi.org/10.1155/2015/303605) show details of* Pseudomonas* and* Mycobacterium* members used in this study.

### 2.2. 16S rRNA and HSP60 Extraction

The files that contain the* Lactobacillus* bacterial genome sequences were processed using Python script to extract each 16S rRNA and HSP60 sequence according to the* Lactobacillus* genome annotations.  Table 2 shows the copy numbers and sequence positions (start-end) of the 16S rRNA and HSP60 sequences in the* Lactobacillus* members and Tables S2 and S4 show the same details of* Pseudomonas* and* Mycobacterium* members used in this study. In one case,* Lactobacillus kefiranofaciens ZW3*, we had to annotate the 16S rRNA sequences, as its annotation was unavailable in the database. We picked up the 16S rRNA sequences from* L. kefiranofaciens ZW3* genome using the same primers successfully used with all other* Lactobacillus* members. The two primers picked up four copies of 16S rRNA sequences (Table 2 strain ID 28).

### 2.3. 16S rRNA Primer Selection

We tested 13 different primer sequences obtained from 8 published studies (Table 3). We used Python script to test the primers and compare the sequence positions we got using each primer with 16S rRNA position in the genome annotation in (NCBI), to confirm that the primer would pick the 16S rRNA sequence. Based on this testing, we selected two primers (Table 3, 8F and 1541R) from [31]. The two selected primers appear in all* Lactobacillus* genomes in this study and with the largest product length (1550 pb).

### 2.4. HSP60 Primer Design

A universal degenerate primer for picking up HSP60 sequences was designed based on the conserved regions in the HSP60 extracted sequences. We identified the conserved regions by performing multiple sequence alignment (MSA) using CLC Sequence Viewer software (CLC Bio, Swansea, UK). Table 3 shows the sequences of the designed and forward and reverse primers.

### 2.5. Restriction Enzymes and Restriction Map

We collected the information about restriction enzymes and restriction sites from the database of restriction enzymes (REBASE), Roberts 1980 and Roberts et al., 2010 [39, 40], and the restriction enzyme database attached to the DNA Star software (DNASTAR Inc., Madison, WI, USA). Prediction of the* in silico* restriction map was performed using the restriction sites information and the seqBuilder tool of Lasergene software tool (DNASTAR Inc., Madison, WI, USA).

## 3. Results and Discussion

### 3.1. Genomics in the Developing Countries

Currently, genome sequencing is the technology-of-choice for several research and clinical applications due to its rapid development, remarkable speed, continuously improved accuracy, and affordable sample processing cost. However, in several developing countries, the genome sequencing technologies are still out of reach for most of researchers and scientists due to several reasons which constrain employing such indispensable technology. Firstly, the high cost of establishing sequencing facility and high cost maintaining the facility in poor-resources countries. Secondly, the lack of well-trained personnel to run the facility. Thirdly, the weak power, Internet, and computational infrastructures. Finally, the limited access to the updated scientific data, literature, and training [26, 27].

The scientific community expected this problem over a decade ago with the rising of the next-generation sequencing technologies [25]. In the following years, many developing countries took steps to utilize these technologies by establishing institutions for genomics and provide funds to facilitate running and maintaining them as well as hiring and training personnel. Reports about case studies in several developing countries including Mexico, Thailand, South Africa, and India show the efforts made to import these technologies and the expected impact on research, public health, and economic development in these countries [41]. Despite these improvements, the problem seems to be still far from being solved, especially in Africa [23, 26], letting the researchers with one choice, that is outsourcing. This situation raises the need of developing alternative methods that can be utilized in doing standard research tasks until the availability of sequencing technologies.

### 3.2. Obtaining Standard Dataset of Bacterial Genomes and Genes

The identification of the family of certain bacteria is usually based on the morphological and other characteristics of the colony, while the identification of the species and strains requires molecular and more sophisticated methods [2, 16, 42, 43]. Therefore, we selected the Lactobacillaceae family as a representative of bacterial population with several industrial and health importance [44–47] to be used in developing FN-Identify method and algorithms. In addition,* Lactobacillus* members have different important genes used in bacterial identification and barcoding such as 16S rRNA and HSP60 with several differences in sequences and copy numbers. This makes* Lactobacillus* members ideal for developing and testing new methods for bacterial identification based on the analysis of the restriction patterns of its genes.

We downloaded the 33 complete* Lactobacillus* genome sequences and annotations available in the NCBI (Table 1). According to the genome annotations, all* Lactobacillus* genomes have one copy from HSP60 and between four and nine copies of 16S rRNA, except for* Lactobacillus kefiranofaciens ZW3* (strain ID: 28, Table 1), where its genome annotation shows absence of 16S rRNA (Table 2). For* Lactobacillus kefiranofaciens ZW3* we annotated the 16S rRNA gene using the selected 16S rRNA universal primers (see below). At least two of 16S rRNA copies are in the reverse direction. Strains under the same species have the same number of 16S rRNA copies except* Lactobacillus johnsonii* strains (strain IDs: 17 and 19, Table 1) since one of them has four copies and the other has six. Tables 1 and 2 list* Lactobacillus* species/strains used in this study as well as the copy numbers, start and end of each copy, and an ID that we gave to each species/strain that we will use hereafter.

### 3.3. Primer Selection and Design

In order to select standard universal primer(s) for 16S rRNA sequences from all* Lactobacillus* genomes, we tested several primers from publish literature (Table 3). We performed the* in silico* screening for each primer using the separated gene sequences as well as the whole genome sequences. Our primers* in silico* screening show that (8F) and (1541R) primers present in most of the separated 16S rRNA gene sequences with largest product length (see Table 3 for primer sequences). Therefore, we keep the sequences between both primers and exclude all other sequences, including the primers sequences.

In some cases, these two primers are not present in 16S rRNA separated sequences. For instance, the two primers failed with the separated 16S rRNA genes of the strain* Lactobacillus salivarius UCC118* (strain ID: 32, Table 1). However, when we used them with the whole genome of the same strain we found 8F and 1541R beginning from nucleotides 74,520 and 76,053, in agreement with the genome annotation of the first 16S rRNA copy (from 74,540 to 76,056). Similarly,* Lactobacillus salivarius CECT 5713* (strain ID: 31, Table 1) has the same difference.

In some cases, there was a difference in length between the 16S rRNA returned* in silico* sequence and the length of the 16S rRNA in the genome annotations. For instance,* Lactobacillus johnsonii* (strain IDs: 17 and 19, Table 1) returned a 1555 bp sequence when using the two selected primers, while the gene length in the genome annotation was 1650 bp. However, it is within the start and end of the annotated gene, so we accept it. Apart from these few cases, the selected 16S rRNA primers 8F and 1541R performed perfectly with all* Lactobacillus* genomes. This guarantees that the returned* in silico* sequences will agree with the isolated sequences in lab.

After selecting the 8F and 1541R primers as universal primers for 16S rRNA, we used them to annotate the 16S rRNA gene in the* Lactobacillus kefiranofaciens ZW3* (strain ID: 28, Table 1) genome. The result shows that the* Lactobacillus kefiranofaciens ZW3* genome contains four copies of 16S rRNA sequences, from nucleotide 125,303 to 126,858 (1555 bp), from 142,446 to 144,001 (1555 bp), from 1,350,707 to 1,352,262 (1555 bp), and from 1,818,440 to 1,819,995 (1555 bp) (Table 2).

For HSP60 gene, we could not find a universal primer in the published literature. Therefore, we design a universal primer based on the conserved nucleotide sequences of HSP60. The conserved nucleotide sequences were identified be multiple sequence alignment (MSA) using CLC Sequence Viewer software (CLC Bio, Swansea, UK). Based on the alignment results, we were able to design two degenerate primers for HSP60 (HSP60-F and HSP60-R, Table 3): the forward primer (HSP60-F) 5′ATGGCWAARGANNTHAARTT3′ and the reverse primer (HSP60-R) 5′TCDGCVACNACNGCTTCNGA3′ yielded in 1560 bp for all species while the annotated HSP60 is 1600 bp. Again, we take the sequences between both primers and exclude all other sequences, including the primers sequences.

### 3.4. *In Silico* Restriction Map

In order to perform an* in silico* enzymatic restriction for the 16S rRNA and HSP60 genes, we selected 12 commercially available restriction enzymes from hundred of enzymes that we collected their data. To select these 12 enzymes, we scanned all enzymes using Python script and the information of the restriction site that we collected from the database of restriction enzymes (REBASE) [40], the restriction enzyme database attached to the DNA Star software (DNASTAR Inc., Madison, WI, USA), and other resources [39], against the 16S rRNA and HSP60 sequences. The selected enzymes have different restriction sites, which will help us differentiate the* Lactobacillus* species through the differences in restriction maps of the selected gene sequences. Next, we performed an* in silico* enzymatic restriction for the 16S rRNA and HSP60 gene sequences using seqBuilder tool of Lasergene software tool (DNASTAR Inc., Madison, WI, USA).

The* in silico* enzymatic digest results in DNA fragment lengths ranges approximately from 10 bp to 1570 bp. Since the very short fragments are unobservable in the experiments, we excluded the fragments length less than 30 bp [48]. Although it is expected that the number of return DNA fragments = the number of restriction sites + 1, the results are different from the expected ones and this is mainly due to two reasons: firstly some fragments being equal in length or the difference in lengths being too small to be observed in the gel separation and secondly our exclusion of the very short fragments.

The exclusion of the short fragments was observed in several species and strains from those we used in this study. For instance,* Lactobacillus delbrueckii *subsp.* bulgaricus 2038* (strain ID: 9, Table 1) has six restriction sites for HinfI enzyme but the number of the return DNA fragments was four only. This is because one of the fragments was of length 9 bp, two other fragments are with length of 119, and two other fragments are with very close length (difference is less than 10 bp) [49]. The same strain has five restriction sites for TfiI but the return DNA fragments contain one fragment of length 9 bp. Therefore, it returns five fragments only. Table S5 contains the details of the return DNA fragments for each restriction enzyme.

Other sources of differences in ribotyping between the* Lactobacillus* genomes are the variation in the 16S rRNA copy numbers between different species and the differences in sequences between the multiple copies within the same genome (Table 2). This leads to difference in restriction sites and number of restriction fragments. One noticeable example for this phenomenon is the* Lactobacillus brevis ATCC 367* (strain ID: 5, Table 1), which contains five copies of 16S rRNA genes with three different sequences (Table 2). The restriction of these three different sequences with HinfI enzyme results in four, five, and six DNA fragments since they have three, four, and five HinfI restriction sites, respectively (Table S5). The same three different sequences of 16S rRNA contain two, three, and three restriction sites for TfiI enzyme, respectively. Another example is* Lactobacillus fermentum* (strain IDs: 12 and 13) that shows similar behavior with the HinfI enzyme (Table 2 and Table S5)

To determine the number of returned DNA fragment from a particular species/strain that contains several copies of 16S rRNA sequences, we compare the lengths of the fragments and exclude the duplicated equal fragments length. This is how the restriction will be done actually in the lab, as the fragments with the same length will be in the same band in the gel. For instance, for* Lactobacillus brevis ATCC 367* (strain ID: 5) there are five different copies of 16S rRNA with three different sequences (see above) (Table 2). Restriction with HinfI enzyme returned five fragments for two copies and four for the other copy. After excluding the duplicated fragment lengths, we have seven fragments only in the gel (976 bp, 891 bp, 379 bp, 243 bp, 136 bp, 117 bp, and 86 bp). Supplementary Figure 1 shows comparison of two cases where he fragments number is equal to the expected and where it is not.

For HSP60 gene, the construction of the restriction map was straightforward. Each* Lactobacillus* species or strain contains one single copy of the gene (Table 2). Therefore, the differentiation between them will be based on differences in restriction patterns between species/strains (Table S7).

## 4. FN-Identify Method

This section describes our proposed sequencing-free bacterial identification method in detail. The proposed method identifies bacterial species/strains based on the number of fragments and/or fragment lengths that result from the restriction of certain genes using a given set of restriction enzymes. Therefore, we refer to it as the fragment number-identification method or FN-Identify. The main goal of FN-Identify is to establish an identification scheme for bacterial species utilizing fragments patterns of enzymatic restrictions such as the restriction map we built in the above section. The established scheme specifies the set of enzymes that could be employed to identify a given (unknown) gene sequence as well as the order of their application. The identified gene refers to a particular species/strain within the restriction map.

The idea behind FN-Identify is inspired from two basic observations. First, the number of fragments resulting from each restriction of a DNA sequence (e.g., 16S rRNA gene sequence) would differ based on the employed restriction enzyme. Generally speaking, a given gene sequence *𝒢* could be split into *m*_*i*_ and *m*_*j*_ fragments if two different enzymes, *e*_*i*_ and *e*_*j*_, were employed, respectively, where *m*_*i*_ and *m*_*j*_ are likely to be different. Second, some restriction enzymes are more discriminative than other enzymes with respect to different bacterial families. Assume that both *e*_*i*_ and *e*_*j*_ are employed to cut all sequences belonging to a specific bacterial family *ℱ*. Let *N*_*e*_*k*__ be a set containing the number of fragments resulting from cutting all sequences of *ℱ* using *e*_*k*_. Enzyme *e*_*i*_ is said to be more discriminative than *e*_*j*_ if and only if |*N*_*e*_*i*__| > |*N*_*e*_*j*__|, where |*A*| denotes the cardinality of set *A*.

For the purpose of illustration, consider an extreme example where all sequences of *ℱ* are split into the same number of fragments if *e*_*j*_ is employed, that is, |*N*_*e*_*j*__| = 1, whereas each sequence of *ℱ* produces a different number of sequences if *e*_*i*_ is employed; that is, |*N*_*e*_*i*__| = *n*, where *n* is the number of sequences of *ℱ*. Clearly, while *e*_*i*_ can identify all sequences of *ℱ* perfectly, *e*_*j*_ does not provide any useful information for discriminating the sequences of *ℱ*. FN-Identify benefits from the above two observations to create an identification scheme for bacterial genes utilizing only a set of discriminating restriction enzymes. The proposed method consists of two algorithms. The first algorithm,* CreateScheme*, aims at finding an efficient identification scheme given a bacterial family *ℱ* and the adopted set of restriction enzymes *ℰ*. The second algorithm,* GeneIdentify*, employs the obtained scheme to identify a given unknown gene sequence.

**Figure 2:**
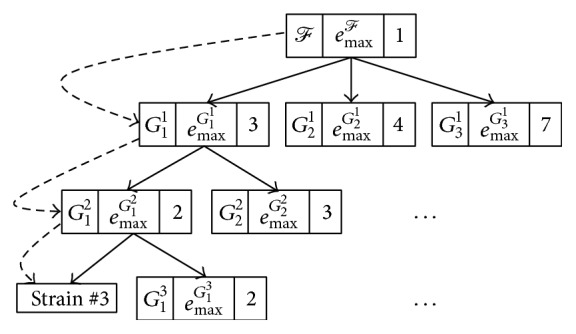
An example of a tree *T* representing an identification scheme. Dotted lines points to a strain that is identified.

The* CreateScheme* algorithm (see Algorithm 1) recursively builds a tree *T* that represents an identification scheme for species/strains of *ℱ*. Each node of *T* consists of three components, namely, the processed group of species/strains, the restriction enzyme that produces the largest number of distinct number of fragments when applied to that group, and the number of distinct number of fragments produced. Obviously, the first component of the root node of *T* (Figure 2) consists of all species/strains of *ℱ* and the third component should be 1 since all species/strains of *ℱ* consist of only one fragment, that is, the whole sequence. Once the enzyme that produces the largest number of distinct number of fragments, for all members of *ℱ*, is found, it should be assigned to the second component of root(*T*). Algorithm 1 can be described informally as follows.

**Step 1 .**

Predict the restriction map *ℛℳ* by restricting all species/strains of *ℱ* using all enzymes of *ℰ*.

**Step 2 .**

Search *ℛℳ* to find the restriction enzyme *e*_max_^*ℱ*^ that gives the largest number of distinct number of fragments when applied to all the gene sequences of *ℱ*.

**Step 3 .**

Use results obtained from the application of *e*_max_^*ℱ*^ to assemble species/strains of *ℱ* into different groups according to the resulting number of fragments such that strain sequences that are split into the same number of fragments are grouped together in the same category. As an example, Figure 2 shows that the restriction-enzyme *e*_max_^*ℱ*^ categorizes the species/strains of *ℱ* into three different groups, namely, *G*_1_^1^, *G*_2_^1^, and *G*_3_^1^, where the superscript indicates the tree level (level 1). All species/strains in these groups are fragmented, using *e*_max_^*ℱ*^, into 3, 4, and 7 fragments, respectively.

**Step 4 .**

Apply Step 3 recursively to each resulting group consisting of more than one species/strains. For example, the illustrative example in Figure 2 shows that the first group in level 1, *G*_1_^1^, is then split into two different groups, *G*_1_^2^ and *G*_2_^2^ in level 2, where species/strains in these groups are fragmented into two and three fragments employing the restriction enzyme *e*_max_^*G*_1_^1^^, respectively.

**Step 5 .**

The algorithm stops if either (1) the number of species/strains of all groups being processed is one or (2) no further application of any restriction enzyme can discriminate species/strains in groups containing more than one species/strains. The former case indicates that the algorithm can identify all species/strains of *ℱ* using the adopted set of restriction enzymes *ℰ*. The second case, on the other hand, takes place if some species/strains cannot be identified employing *ℰ*. In this case, another factor, such as the fragment length, can be utilized to break any potential ties among unidentified species/strains.

Once an identification scheme *T* is created for *ℱ*, it would be possible to identify an unknown gene sequence *𝒢* as belonging to *ℱ* or not by traversing *T* starting from the root node following the* GeneIdentify* algorithm (see Algorithm 2). The first step is to visit the root node of *T* to specify the restriction enzyme that should be employed first to cut *𝒢*, that is, *e*_max_^*ℱ*^. Then, the number of fragments of all children (groups) of the current node (root) is retrieved and compared to the number of fragments resulting from cutting *𝒢* using *e*_max_^*ℱ*^. The node of the matched group is then visited and its associated restriction enzyme is retrieved and applied to *𝒢* in order to decide which node has to be visited in the next level, and so on. This process is continued until a leaf node is met. If such a node is found, the processed gene sequence will be successfully identified as the species/strains at that (leaf) node. Otherwise, the identification process fails. As mentioned earlier, if there are no matching groups at any level of *T*, a different factor such as lengths of fragments could be tried and the identification process will continue.

The* GeneIdentify* algorithm can be illustrated further using the example shown in Figure 2. Let a strain *𝒢* be one of the strains, referred to as* strain* #3, that belongs to *ℱ*. In this example (see dashed lines), *𝒢* is identified by applying the following sequence of restriction enzymes: *e*_max_^*ℱ*^, *e*_max_^*G*_1_^1^^, and *e*_max_^*G*_1_^2^^. This is because *𝒢* is split into three fragments if *e*_max_^*ℱ*^ is employed and two fragments if *e*_max_^*G*_1_^1^^ is employed and no other species/strain is fragmented into the same number of fragments if *e*_max_^*G*_1_^2^^ is employed to cut *ℱ*.

### 4.1. Developing FN-Identify Method

**Figure 3:**
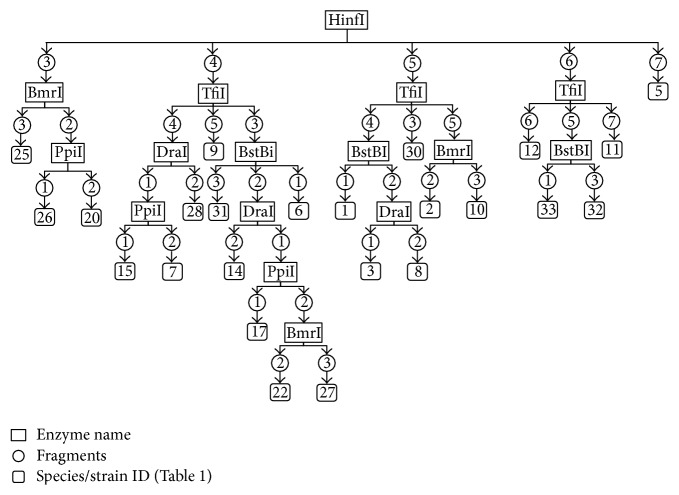
Identification scheme of* Lactobacillus* using the fragments numbers only of the 16S rRNA gene, proposed by FN-Identify.

**Figure 4:**
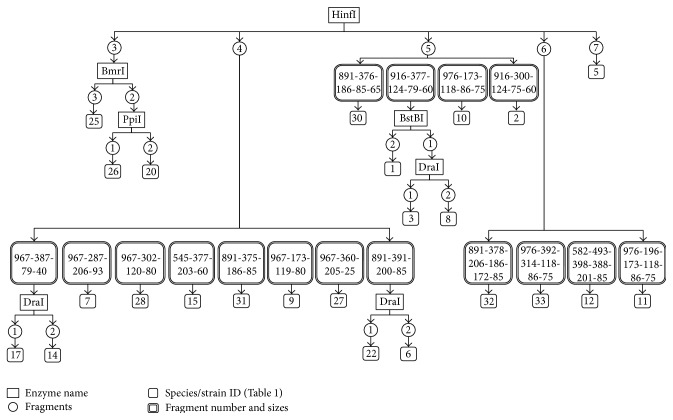
Identification scheme of* Lactobacillus* using the fragments numbers and fragments lengths of the 16S rRNA gene, proposed by FN-Identify.

In order to develop our proposed method and algorithms, we used the 16S rRNA sequences of a population of 33 members of* Lactobacillus* (Table 1), an example of bacteria with genes with multiple copies in the genome (Table 2). FN-Identify and the two algorithms were able to identify and differentiate between the 33 species/strains based on the fragment numbers of the 16S rRNA sequences using six restriction enzymes (Figure 3, Supplementary Table 5). For a given species/strains a minimum of one enzyme and maximum of five enzymes were required for the identification (Figure 3 strains ID: 5 and 8, resp.). By adding the fragment length as a second factor, FN-Identify successfully identify and differentiate between the 33 species/strains using five restriction enzymes only. Furthermore, a maximum of three enzymes only was required for the identification of any given species/strains (Figure 4, Supplementary Table 6).

To further improve the identification efficiency of FN-Identify method and algorithms, we used the HSP60 genes as an example for genes with a single copy in the genome (Table 2). Genes represented with a single copy provide less variations in sequences (see above). Therefore FN-Identify might require more restriction enzymes to differentiate the 33 species/strains or even may fail in identifying some of them. However, with further tuning of the algorithms, FN-Identify shows comparable performance to what it does in the genes represented with a multiple copies (16S rRNA). It was able to identify the 33 species/strains based on the fragment numbers using six restriction enzymes (Supplementary Figure 2 and Supplementary Table 7). For a given species/strains a minimum of two enzymes and maximum of five enzymes were required for the identification (Supplementary Figure 2 strains ID: 24 and 33, resp.). When we used the fragment length as a second factor, FN-Identify required four restriction enzymes only to identify the 33 species/strains (Supplementary Figure 3 and Supplementary Table 8). Moreover, a maximum of three enzymes only was required for the identification of a given species/strains (Table 4, Supplementary Figure 3, and Supplementary Table 8). In some cases, the gene sequences and copy numbers of two strains are the same. Therefore neither FN-Identify nor the sequencing-based approach can differentiate them, such as strains* Lactobacillus rhamnosus ATCC 53103* and* Lactobacillus rhamnosus GG* (Table 1, strain IDs: 24 and 25) (Figures 3 and 4).

### 4.2. Testing and Assessment of FN-Identify Method

FN-Identify method and the two algorithms were developed using a training set of 33 members of* Lactobacillus* with two sets of gene sequences (16S rRNA and HSP60). To test FN-Identify method and algorithms performance, we assessed its identification efficiency using two different testing sets from two distinct bacterial groups* Mycobacterium* and* Pseudomonas*.* Mycobacterium* is a Gram-positive bacterial genus from the Mycobacteriaceae family that includes members that cause serious illness such as* Mycobacterium tuberculosis*, which causes tuberculosis.* Pseudomonas* is a Gram-negative bacterial genus from the Pseudomonadaceae that includes important model organisms such as* Pseudomonas aeruginosa*.

We obtained the sequences of the 16S rRNA genes of 22 members of* Mycobacterium* and 33 members of* Pseudomonas* using the same approach that we used with* Lactobacillus* (See Section 2). The variations in the 16S rRNA copy number and differences sequences between the multiple copies within the same genome appear in* Pseudomonas*, whereas the* Mycobacterium* genomes of the 22 members contain only one or two 16S rRNA copies (Supplementary Tables 2 and 4). We applied FN-Identify on the two testing datasets and FN-Identify successfully identified all the members of the two groups using the fragments numbers only and eight and seven enzymes to identify the 33 members of* Pseudomonas* and the 22 members of* Mycobacterium*, respectively (Table 4). Furthermore, for a given species, a maximum of eight and seven enzymes and minimum of seven and five enzymes were required to identify a given member of the* Pseudomonas* and* Mycobacterium* groups, respectively (Table 4, Supplementary Figures 4 and 6, and Supplementary Tables 9 and 11). By adding the fragment length as a second factor, FN-Identify successfully identifies the species/strains of the two groups using seven and four restriction enzymes for* Pseudomonas* and* Mycobacterium* groups, respectively. Furthermore, a maximum of seven and four enzymes and a minimum of four and three enzymes were required for the identification of any given species/strains (Table 4, Supplementary Figures 5 and 7, and Supplementary Tables 10 and 12).

Collectively, these results demonstrate the efficiency and utility of the FN-Identify method and the two developed algorithms in identifying bacterial species/strains within a genus and show that the method is applicable in bacterial groups with distinct properties.

### 4.3. Applications and Future Perspective

The assessment of FN-Identify method and the two developed algorithms shows the potentials of the method, with standard microbiology protocols and instruments. FN-Identify is a computational method that is designed as an aid that helps designing and minimizing the experimental procedures required for bacterial identification. Ideally, FN-Identify interfaces with the experimental and clinical workflows through receiving inputs (expected bacterial group, gene(s) to be used for identification, and list of restriction enzymes) and provides outputs that lead the later bench exterminates (list and order of enzymatic restriction experiments and the identification scheme that is used to interpret the experimental results).

To be fully utilized, FN-Identify needs a software tool that is connected with a database of gene sequence (e.g., 16S rRNA and HSP60) in different bacterial families and database of restriction enzymes. The software should implement the two algorithms and automate the selection of the species and the enzymes as well as automating building the restriction map and the identifying scheme. We are currently building this tool as a webserver that provides these services for free to enable the scientific community in the developing countries to utilize FN-Identify.

## 5. Conclusion

Bacterial identification is an important routine that is required in several microbiological and environmental applications and research. The current techniques are highly dependent on genome sequencing techniques that target certain genes that present almost in all bacterial species. Although the genome sequencing techniques observed outstanding improvements in accuracy and decrease in cost, developing countries remain far from employing these indispensable technologies due to several barriers. Therefore, alternative sequencing-independent methods are required to facilitate the needed tasks with affordable costs and using the available facilities. We developed FN-Identify method, a sequencing-independent method for bacterial identification, using standard microbiological protocols and instruments, restriction enzymes, and two algorithms that we developed (CreateScheme and GeneIdentify). FN-Identify was tested against standard bacterial populations of 22 and 33 bacterial species/strains of the* Mycobacterium* and* Pseudomonas* groups, respectively. The method successfully differentiate all the species/strains in two independent analyses using two different genes 16S rRNA and HSP60 for each of the two groups. A webserver is being developed for FN-Identify to automate the scheme building and maximize the utilization of the method. We believe that FN-Identify is a useful alternative to the sequencing methods when they are out of reach.

## Supplementary Material

The supplementary materials include seven supplementary figures and 12 supplementary tables. Supplementary figure 1 is an illustration of expected restriction results of two *Lactobacillus* strains. Supplementary figures 2 and 3 are the Identification schemes of *Lactobacillus* using fragments numbers or fragments numbers and fragments size of HSP60 gene. Supplementary figures 4-7 are the Identification schemes of *Pseudomonas* and *Mycobacterium* using fragments numbers only or fragments number and fragments size of 16S RNA gene. Supplementary tables 1-4 list the details of species and strains of *Pseudomonas* and *Mycobacterium* that used in this study. Supplementary tables 5-12 are the restriction maps of the species and strains of *Lactobacillus*, *Pseudomonas* and *Mycobacterium* used as input to FN-Identify.

## Figures and Tables

**Algorithm 1:**
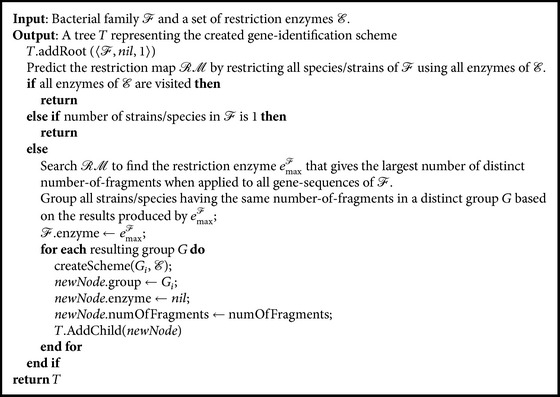
*CreateScheme*(*ℱ*, *ℰ*).

**Algorithm 2:**
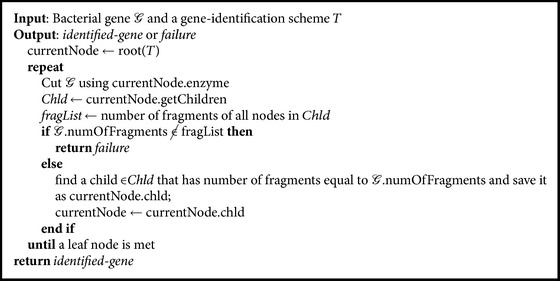
*GeneIdentify*(*𝒢*, *T*).

**Table 1:** Names and GenBank accession number of *Lactobacillus* species used in this study.

Strain ID^*∗*^	Organism	GenBank accession number
1	*Lactobacillus acidophilus 30SC*	CP002559
2	*Lactobacillus acidophilus NCFM*	CP000033
3	*Lactobacillus amylovorus GRL 1112*	CP002338
4	*Lactobacillus amylovorus GRL 1118*	CP002609
5	*Lactobacillus brevis ATCC 367*	CP000416
6	*Lactobacillus buchneri NRRL B-30929*	CP002652
7	*Lactobacillus casei ATCC 334*	CP000423
8	*Lactobacillus crispatus ST1*	FN692037
9	*Lactobacillus delbrueckii* subsp. *bulgaricus 2038*	CP000156
10	*Lactobacillus delbrueckii* subsp. *bulgaricus ATCC 11842*	CR954253
11	*Lactobacillus delbrueckii* subsp. *bulgaricus ATCC BAA-365*	CP000412
12	*Lactobacillus fermentum CECT 5716*	CP002033
13	*Lactobacillus fermentum IFO 3956*	AP008937
14	*Lactobacillus gasseri ATCC 33323*	CP000413
15	*Lactobacillus helveticus DPC 4571*	CP000517
16	*Lactobacillus helveticus H10*	CP002429
17	*Lactobacillus johnsonii DPC 6026*	CP002464
18	*Lactobacillus johnsonii FI9785*	FN298497
19	*Lactobacillus johnsonii NCC 533*	AE017198
20	*Lactobacillus plantarum JDM1*	CP001617
21	*Lactobacillus plantarum* subsp. *plantarum ST-III*	CP002222
22	*Lactobacillus reuteri DSM 20016*	CP000705
23	*Lactobacillus reuteri JCM 1112*	AP007281
24	*Lactobacillus rhamnosus ATCC 53103*	AP011548
25	*Lactobacillus rhamnosus GG*	FM179322
26	*Lactobacillus rhamnosus Lc 705*	FM179323
27	*Lactobacillus sakei 23K*	CR936503
28	*Lactobacillus kefiranofaciens ZW3*	CP002764
29	*Lactobacillus Paracasei 8700:2*	CP002391
30	*Lactobacillus ruminis ATCC 27782*	CP003032
31	*Lactobacillus salivarius CECT 5713*	CP002034
32	*Lactobacillus salivarius UCC118*	CP000233
33	*Lactobacillus sanfranciscensis TMW 1.1304*	CP002461

^*∗*^This ID will be used to refer to the species/strains in the text.

This table lists the studied *Lactobacillus* species/strains and their GenBank accession numbers.

**Table 2:** 16S rRNA and HSP60 copy numbers and genomics positions.

Strain ID	16S rRNA copies number	16S rRNA position	HSP60 position
1	4	57091⋯58665 447399⋯448973 469566⋯471140 1712759⋯1714333	407805⋯409506

2	4	59255⋯60826 413779⋯415350 434247⋯435818 1632689⋯1634260	379688⋯381333

3	4	66295⋯67869 450127⋯451701 469953⋯471527 1743991⋯1745565	403452⋯405083

4	4	55901⋯57475 413067⋯414641 431084⋯432658 1592809⋯1594383	376234⋯377865

5	5	86149⋯87711 453214⋯454776 562993⋯564555 1146802⋯1148364 1504667⋯1506229	645454⋯647079

6	5	706262⋯707824 829466⋯831028 1597799⋯1599360 1678756⋯1680318 2300479⋯2302041	1429276⋯1430898

7	5	259510⋯261077 823779⋯825346 845529⋯847096 1829076⋯1830643 2504379⋯2505946	2233684⋯2235318

8	4	62524⋯64075 427906⋯429457 445456⋯447007 1669931⋯1671482	391450⋯393075

9	9	35825⋯37395 681032⋯682602 789164⋯790734 821185⋯822755 1416360⋯1417930 1526926⋯1528496 1596022⋯1597592 1805404⋯1808393 1818669⋯1820239	1448011⋯1449624

10	9	45160⋯46720 689136⋯690696 806393⋯807953 1359934⋯1361495 1470602⋯1472162 1543296⋯1544856 1576953⋯1578513 1787059⋯1788619 1794646⋯1796206	1392354⋯1393967

11	9	43705⋯45265 683265⋯684825 792486⋯794046 1373565⋯1375125 1483805⋯1485365 1562005⋯1563565 1594263⋯1595823 1792049⋯1793609 1799394⋯1800954	1405173⋯1406786

12	5	169808⋯171375 194092⋯195659 273972⋯275539 651911⋯653482 1564338⋯1565905	394255⋯395886

13	5	169391⋯170958 193655⋯195222 273501⋯275068 651358⋯652925 1563202⋯1564769	393747⋯395378

14	6	477570⋯479148 1559153⋯1560731 1565823⋯1567401 1579997⋯1581575 1786679⋯1788257 1792194⋯1793772	425524⋯427155

15	4	76215⋯77787 450938⋯452510 468198⋯469770 1697386⋯1698958	408372⋯409994

16	4	85110⋯86682 428551⋯430123 446061⋯447633 1736897⋯1738469	393232⋯394854

17	4	546957⋯548607 1653714⋯1655334 1668197⋯1669764 1871317⋯1872967	490210⋯491841

18	4	455618⋯457268 1479559⋯1481209 1494009⋯1495659 1661809⋯1663459	412091⋯413722

19	6	558550⋯560200 1663054⋯1664704 1669721⋯1671371 1684170⋯1685820 1882821⋯1884471 1888336⋯1889986	502509⋯504140

20	5	484838⋯486408 1155088⋯1156658 1985568⋯1987138 2410113⋯2411683 2860684⋯2862254	631044⋯632669

21	5	487643⋯489213 1132007⋯1133577 1988715⋯1990285 2469054⋯2470624 2918612⋯2920182	591466⋯593091

22	6	177728⋯179296 312393⋯313961 624382⋯625950 639563⋯641131 1077760⋯1079328 1373427⋯1374995	401807⋯403435

23	6	177347⋯178880 312212⋯313745 632685⋯634218 649117⋯650650 1117409⋯1118942 1412879⋯1414412	401630⋯403258

24	5	306772⋯308345 820809⋯822382 840850⋯842423 1923809⋯1925382 2563756⋯2565329	2303140⋯2304732

25	5	307756⋯309313 823249⋯824806 843290⋯844847 1929410⋯1930967 2568485⋯2570042	2308734⋯2310368

26	5	289782⋯291339 817799⋯819356 837823⋯839380 1895692⋯1897249 2548360⋯2549917	2265733⋯2267367

27	7	306178⋯307748 445757⋯447106 478891⋯480461 1575575⋯1577145 1762644⋯1763993 1867063⋯1868633 1872479⋯1873828	358686⋯360625

28	4^1^	125303⋯126858 142446⋯144001 1350707⋯1352262 1818440⋯1819995	82036⋯83667

29	5	274946⋯276503 774656⋯776213 794023⋯795580 1866160⋯1867717 2503645⋯2505202	2240006⋯2241640

30	6	274311⋯275837 393951⋯395477 449057⋯450583 759032⋯760558 1507426⋯1508592 1947545⋯1949071	650101⋯651714

31	7	74995⋯76521 218268⋯219794 435427⋯436953 480965⋯482491 1301435⋯1302951 1411138⋯1412654 1818075⋯1819591	1247027⋯1248649

32	7	74540⋯76056 217778⋯219294 434853⋯436380 480393⋯481909 1300792⋯1302308 1410454⋯1411970 1817320⋯1818824	1246385⋯1248007

33	7	40703⋯42272 121127⋯122696 360538⋯362108 367314⋯368884 422087⋯423657 1008778⋯1010348 1279132⋯1280701	485966⋯487585

^1^Our Annotation for 16S rRNA sequences in *L. kefiranofaciens ZW3*.

**Table 3:** Primer sequences used for 16S rRNA.

ID	Gene name	Name	Sequence	Reference
1	16S rRNA	8F^*∗*^	5′AGAGTTTGATCCTGGCTC AG3′	[31]
2	16S rRNA	U1492R	5′GGTTACCTTGTTACGACTT3′	[32]
3	16S rRNA	928F	5′TAAAACTYAAAKGAATTGACGGG3′	[33]
4	16S rRNA	336R	5′ACTGCTGCSYCCCGTAGGAGTCT3′	[33]
5	16S rRNA	1100F	5′YAACGAGCGCAACCC3′	[34]
6	16S rRNA	1100R	5′AGGGTTGCGCTCGTTG3′	[34]
7	16S rRNA	907R	5′CCGTCAATTCCTTTRAGTTT3′	[34]
8	16S rRNA	785F	5′GGATTAGATACCCTGGTA3′	[35]
9	16S rRNA	805R	5′GACTACCAGGGTATCTAATC3′	[36]
10	16S rRNA	515F	5′GTGCCAGCMGCCGCGGTAA3′	[34]
11	16S rRNA	518R	5′GTATTACCGCGGCTGCTGG3′	[37]
12	16S rRNA	27F	5′AGAGTTTGATCMTGGCTCAG3′	[38]
13	16S rRNA	1541R^*∗*^	5′AAGGAGGTGATCCAGCCGCA3′	[31]
14	HSP60	HSP60-F	5′ATGGCWAARGANNTHAARTT3′	Designed
15	HSP60	HSP60-R	5′TCDGCVACNACNGCTTCNGA3′	Designed

^*∗*^16S rRNA selected primers.

**Table 4:** Summary of the employed training and testing datasets and FN-Identify performance.

Bacterial group	Gram^1^	Members	16S rRNA					HSP60				
			Unique sequences^2^	Required enzymes		Max.-Min. Enzymes/species^3^		Unique sequences^2^	Required enzymes		Max.-Min. Enzymes/species^3^	
				1 factor	2 factors	1 factor	2 factors		1 factor	2 factors	1 factor	2 factors
Training set												
*Lactobacillus*	P.	33	24	6	5	6-6	5-3	23	6	5	4-1	3-1
Testing sets												
*Pseudomonas*	N.	33	32	8	6	8-7	7-4	—	—	—	—	—
*Mycobacterium*	P.	22	18	7	4	7-5	4-3	—	—	—	—	—

^1^P: positive and N: negative.

^2^Members with differences in 16S rRNA sequences. In some cases two or more members have 100% similarity in 16S rRNA sequences. Those members are considered as one entry to FN-Identify.

^3^The maximum and minimum number of enzymes required identifying a given member of the group.
