# Supplementary material for: FN-Identify: Novel Restriction Enzymes-Based Method for Bacterial Identification in Absence of Genome Sequencing
Source: Adv Bioinformatics. 2015 Dec 31;2015:303605. doi: 10.1155/2015/303605 (PMC4735980; doi:10.1155/2015/303605)

Supplementary Figure 1

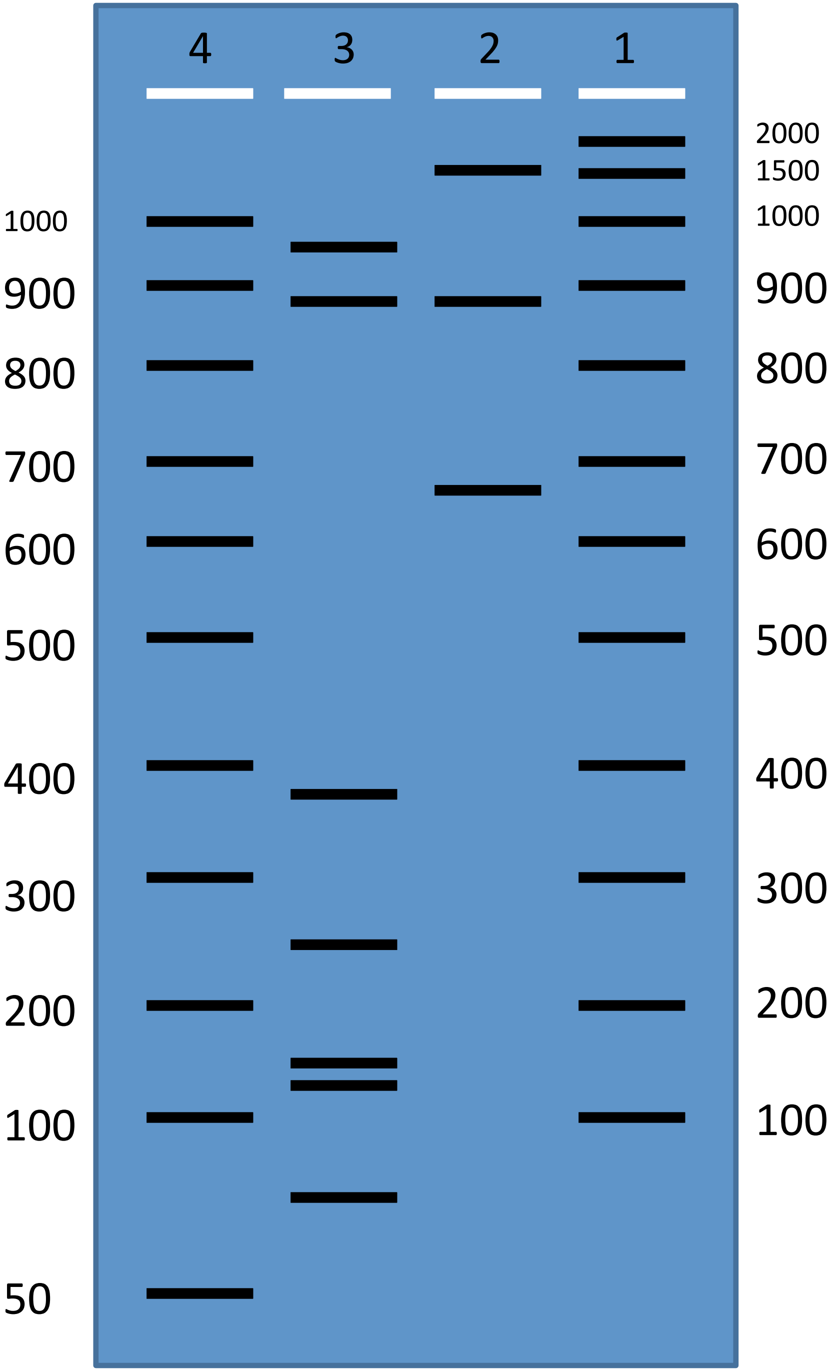

Supplementary Figure 2

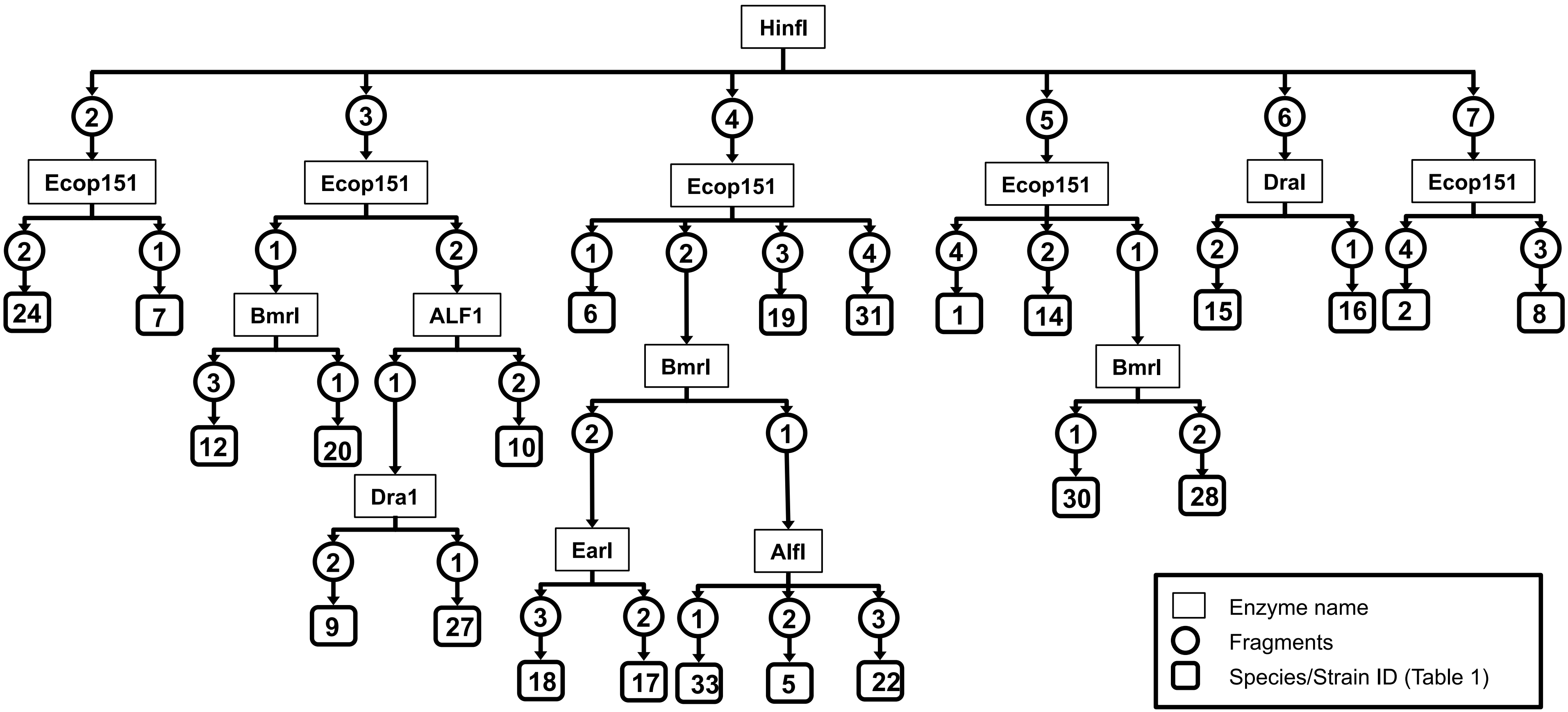

Supplementary Figure 3

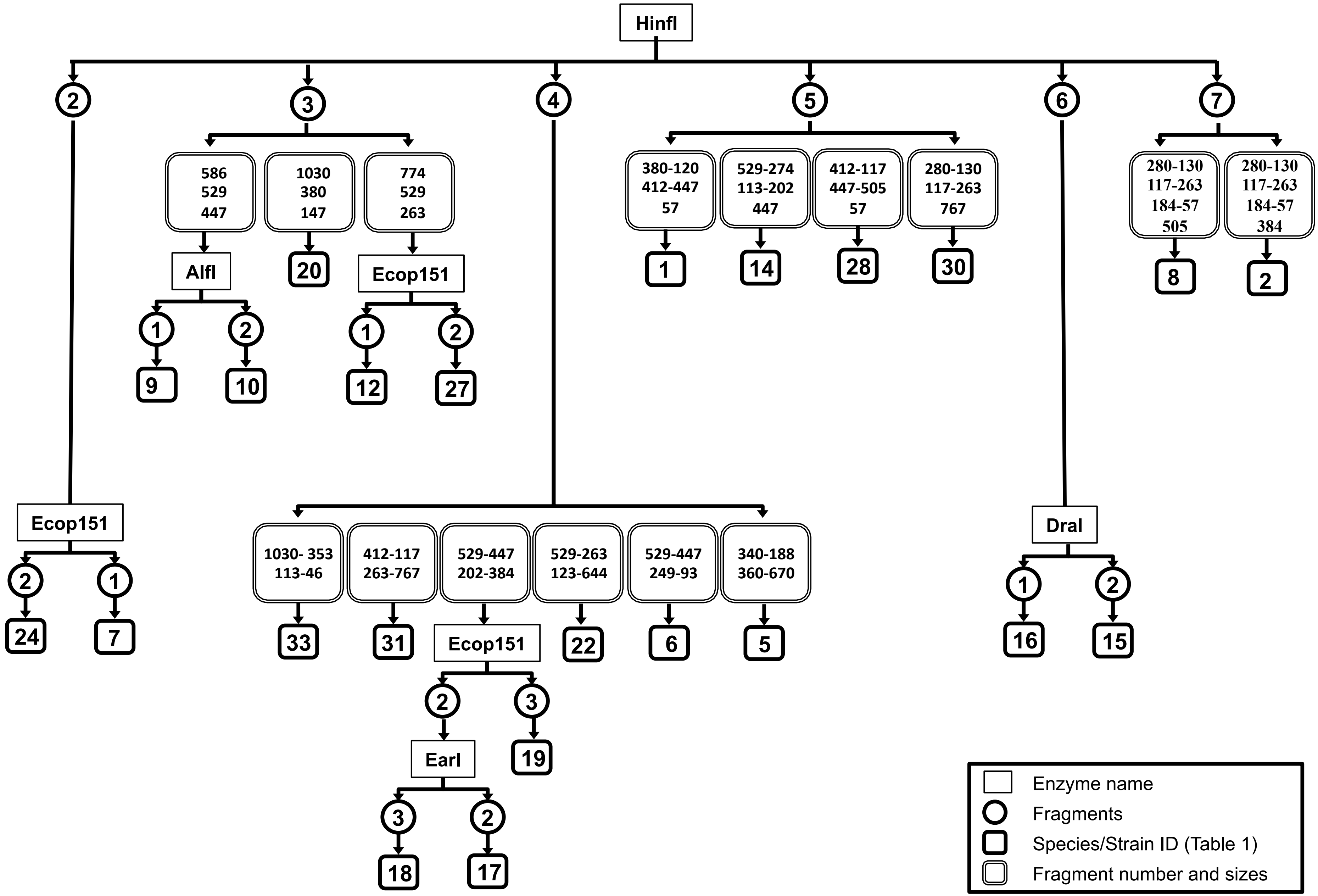

Supplementary Figure 4

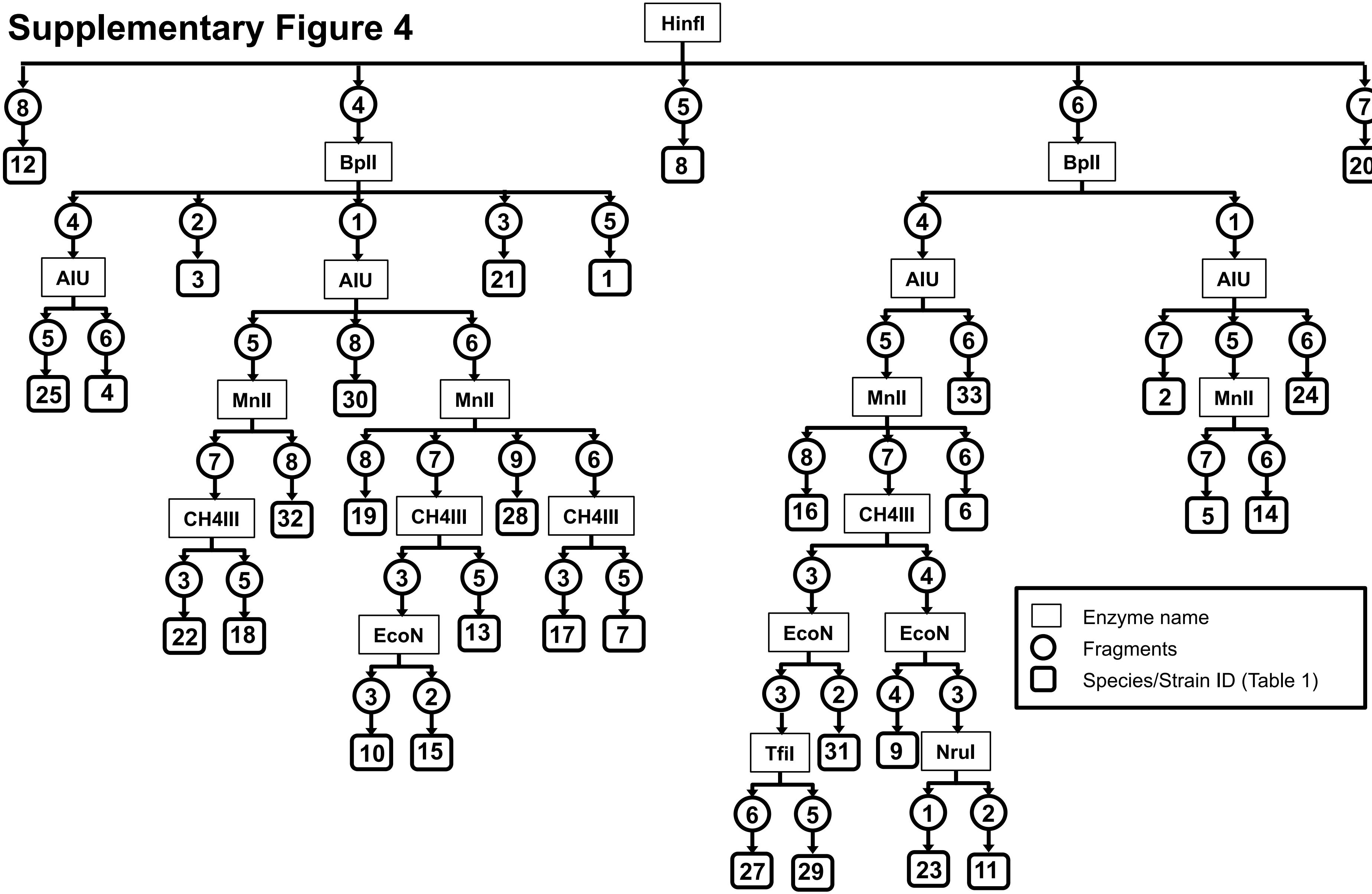

Supplementary Figure 5

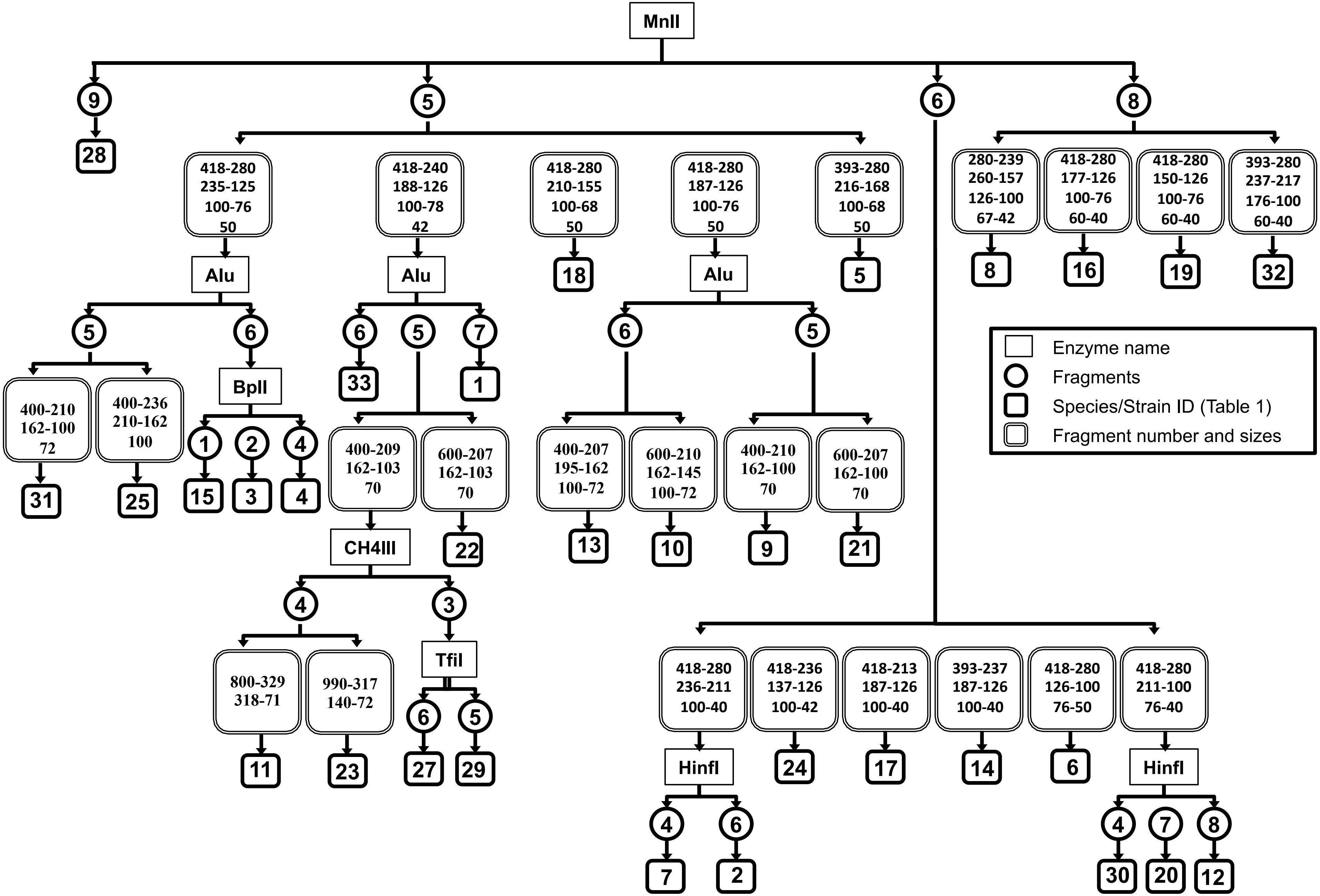

Supplementary Figure 6

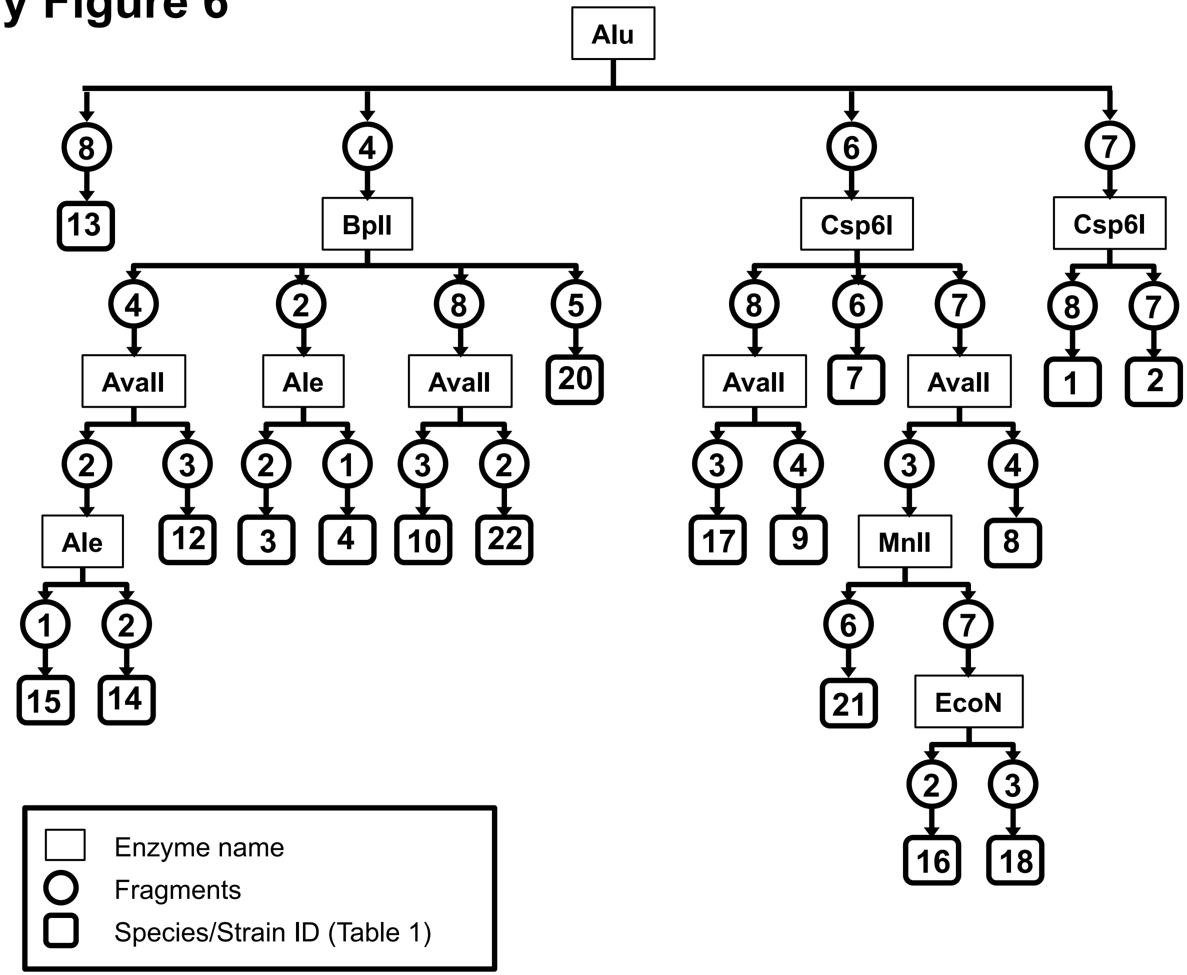

Supplementary Figure 7

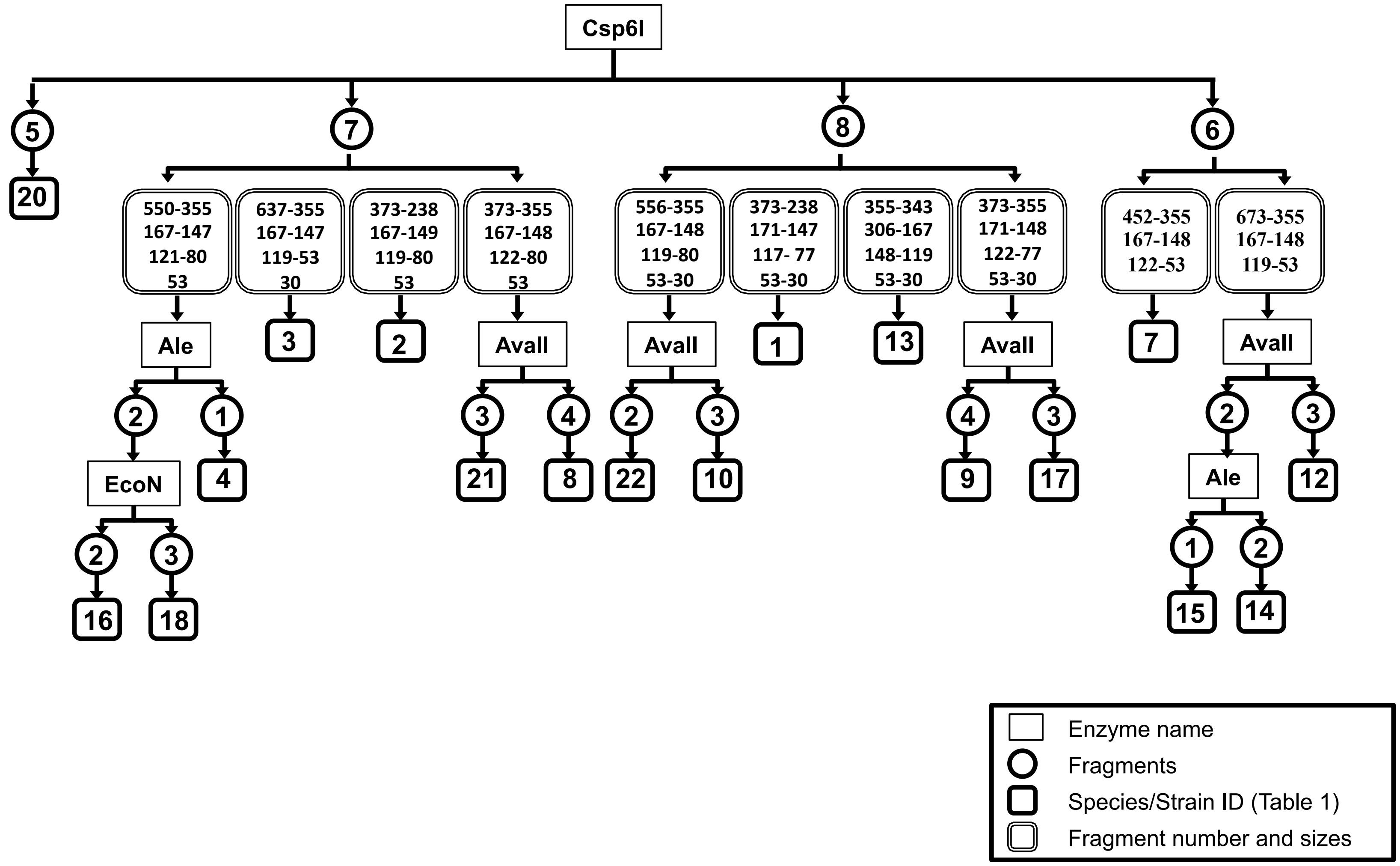

Supplement: Supplementary file 1 — The supplementary materials include seven supplementary figures and 12 supplementary tables. Supplementary figure 1 is an illustration of expected restriction results of two Lactobacillus strains. Supplementary figures 2 and 3 are the Identification schemes of Lactobacillus using fragments numbers or fragments numbers and fragments size of HSP60 gene. Supplementary figures 4-7 are the Identification schemes of Pseudomonas and Mycobacterium using fragments numbers only or fragments number and fragments size of 16S RNA gene. Supplementary tables 1-4 list the details of species and strains of Pseudomonas and Mycobacterium that used in this study. Supplementary tables 5-12 are the restriction maps of the species and strains of Lactobacillus, Pseudomonas and Mycobacterium used as input to FN-Identify. [file 303605.f1.zip › Awad-etal-SupplementaryFigures.pdf]
