# Supplementary material for: FN-Identify: Novel Restriction Enzymes-Based Method for Bacterial Identification in Absence of Genome Sequencing
Source: Adv Bioinformatics. 2015 Dec 31;2015:303605. doi: 10.1155/2015/303605 (PMC4735980; doi:10.1155/2015/303605)
Supplement: Supplementary file 1 — The supplementary materials include seven supplementary figures and 12 supplementary tables. Supplementary figure 1 is an illustration of expected restriction results of two Lactobacillus strains. Supplementary figures 2 and 3 are the Identification schemes of Lactobacillus using fragments numbers or fragments numbers and fragments size of HSP60 gene. Supplementary figures 4-7 are the Identification schemes of Pseudomonas and Mycobacterium using fragments numbers only or fragments number and fragments size of 16S RNA gene. Supplementary tables 1-4 list the details of species and strains of Pseudomonas and Mycobacterium that used in this study. Supplementary tables 5-12 are the restriction maps of the species and strains of Lactobacillus, Pseudomonas and Mycobacterium used as input to FN-Identify. [file 303605.f1.zip › Awad-etal-SupplementaryTable1.docx]

**Supplementary Table 1: Names and GenBank accession number of *Pseudomonas* species used in this study**

| **Strain**  **ID*** | **Organism** | **GenBank**  **Accession**  **Number** | **Strain**  **ID*** | **Organism** | **GenBank**  **Accession**  **Number** |
| --- | --- | --- | --- | --- | --- |
| 1 | *Pseudomonas syringae pv. syringae* | CP000075 | 18 | *Pseudomonas resinovorans* | AP013068 |
| 2 | *Pseudomonas stutzeri* | CP000304 | 19 | *Pseudomonas chlororaphis* | CP011110 |
| 3 | *Pseudomonas savastanoi pv. savastanoi* | CM001834 | 20 | *Pseudomonas denitrificans* | CP004143 |
| 4 | *Pseudomonas syringae pv. phaseolicola* | CP000058 | 21 | *Pseudomonas poae RE*1-1-14* | CP004045 |
| 5 | *Pseudomonas aeruginosa* | AE004091 | 22 | *Pseudomonas fluorescens* | AM181176 |
| 6 | *Pseudomonas entomophila* | CT573326 | 23 | *Pseudomonas mosselii* | CP009365 |
| 7 | *Pseudomonas pseudoalcaligenes* | HG916826 | 24 | *Pseudomonas moraviensis* | CM002330 |
| 8 | *Pseudomonas syringae pv. Tomato* | AE016853 | 25 | *Pseudomonas cichorii* | CP007039 |
| 9 | *Pseudomonas putida* | AE015451 | 26 | *Pseudomonas protegens* | CP000076 |
| 10 | *Pseudomonas synxantha* | CM001514 | 27 | *Pseudomonas alkylphenolia* | CP009048 |
| 11 | *Pseudomonas monteilii* | CP006978 | 28 | *Pseudomonas simiae* | CP007637 |
| 12 | *Pseudomonas knackmussii* | HG322950 | 29 | *Pseudomonas rhizosphaerae* | CP009533 |
| 13 | *Pseudomonas brassicacearum* | CP007410 | 30 | *Pseudomonas balearica* | CP007511 |
| 14 | *Pseudomonas fulva 12-X* | CP002727 | 31 | *Pseudomonas cremoricolorata* | CP009455 |
| 15 | *Pseudomonas mandelii* | CP005960 | 32 | *Pseudomonas mendocina ymp* | CP000680 |
| 16 | *Pseudomonas parafulva* | CP009747 | 33 | *Pseudomonas plecoglossicida* | CP010359 |
| 17 | *Pseudomonas protegens* | AP014522 |  |  |  |
