# Supplementary material for: FN-Identify: Novel Restriction Enzymes-Based Method for Bacterial Identification in Absence of Genome Sequencing
Source: Adv Bioinformatics. 2015 Dec 31;2015:303605. doi: 10.1155/2015/303605 (PMC4735980; doi:10.1155/2015/303605)
Supplement: Supplementary file 1 — The supplementary materials include seven supplementary figures and 12 supplementary tables. Supplementary figure 1 is an illustration of expected restriction results of two Lactobacillus strains. Supplementary figures 2 and 3 are the Identification schemes of Lactobacillus using fragments numbers or fragments numbers and fragments size of HSP60 gene. Supplementary figures 4-7 are the Identification schemes of Pseudomonas and Mycobacterium using fragments numbers only or fragments number and fragments size of 16S RNA gene. Supplementary tables 1-4 list the details of species and strains of Pseudomonas and Mycobacterium that used in this study. Supplementary tables 5-12 are the restriction maps of the species and strains of Lactobacillus, Pseudomonas and Mycobacterium used as input to FN-Identify. [file 303605.f1.zip › Awad-etal-SupplementaryTable2.docx]

**Supplementary table 2: *Pseudomonas* 16S rRNA copy numbers and positions**

| **Strain**  **ID*** | **16S rRNA**  **Copies** | **16S rRNA**  **Position** | **Strain**  **ID*** | **16S rRNA**  **Copies** | **16S rRNA**  **Position** |
| --- | --- | --- | --- | --- | --- |
| 1 | 5 | 772102..773632  964692..96622  3581141..3852671  5426747..5428258  5951650..5953180 | 18 | 5 | 126338.. 127847 637503.. 639012 3970797.. 3972306 5357066.. 5358575 5362630.. 5364139 |
| 2 | 4 | 866715..868243  1797085..1798613  2534258..2535786  3517465..3518993 | 19 | 5 | 714054.. 715563 1057272.. 1058781 2100133.. 2101642 5823290.. 5824799 6502644.. 6504153 |
| 3 | 1 | 4672185..4673715 | 20 | 5 | 593165.. 594674 2112015.. 2113524 2646750.. 2648259 4665023.. 4666532 5464943.. 5466452 |
| 4 | 5 | 804007..805518  1048742..1050253  3635156..3636667  5250697..5252208  578282..5784337 | 21 | 5 | 1279291.. 1280800 3808306.. 3809815 4136714.. 4138223 4900296.. 4901805 5506894.. 5508403 |
| 5 | 4 | 722102..723610 4792197..4793705 5267725..5269233 6043209..6044717 | 22 | 3 | 119725.. 121234 768537.. 770046 2234932.. 2236441 |
| 6 | 7 | 115498..117007  490039..491548  696507..698016 1375552..1377061 2739320..2740829 3799138..3800647 5002154..5003663 | 23 | 7 | 2966347.. 2967856 3505507.. 3507016 4057771.. 4059280 4131282.. 4132791 5290891.. 5292400 5628576.. 5630085 5838051.. 5839560 |
| 7 | 4 | 1045074..1046583 1756302..1757811 3840503..3842012 4389227..4390736 | 24 | 2 | 670257.. 671765  5372536.. 5374045 |
| 8 | 5 | 666733..668244  827547..829058 1072084..1073595 3873144..3874655 6217615.. 6219126 | 25 | 6 | 735980.. 737491 921582.. 923093 1557598.. 1559109 2030175.. 2031686 5342967.. 5344478 5857987.. 5859498 |
| 9 | 7 | 171378..172887  176808..178317  524938..526447  697813..699322 1325492.. 1327001 2548678..2550187 5311158..5312667 | 26 | 5 | 122817.. 124328 854058.. 855569 4813643.. 4815154 6012595.. 6014106 6386415.. 6387926 |
| 10 | 6 | 144843..146352  816939..818448 2169356..2170865 4756450..4757958 5268392..5269901 5659027..5660536 | 27 | 6 | 154113.. 155622 537087.. 538596 840136.. 841645 1354299.. 1355808 3844122.. 3845631 5093841.. 5095350 |
| 11 | 6 | 125937.. 127446  298090.. 299599 1524849.. 1526358 4268113.. 4269622 4736043.. 4737552 5749753.. 5751262 | 28 | 2 | 2059702.. 2061211 5041202.. 5042711 |
| 12 | 4 | 641273.. 642782 1865895.. 1867404 5180518..5182027 6029332..6030841 | 29 | 6 | 630375.. 631884 2859434.. 2860943 2981922.. 2983431 3518462.. 3519971 4146283.. 4147792 4686261.. 4687770 |
| 13 | 5 | 797303..798812.. 4729572..4731081 5632506..5634015 5988901..5990410 6509085.. 6510594 | 30 | 4 | 1037391.. 1038900 1673322.. 1674833 2240693.. 2242202 3755835.. 3757344 |
| 14 | 4 | 239521.. 241029  691586.. 693094 936927..938435 2802261.. 2803769 | 31 | 6 | 93518.. 95027  520540.. 522049 1564312.. 1565821 1756664.. 1758173 1986806.. 1988315 3179930.. 3181439 |
| 15 | 6 | 1654527.. 3114933 3116443.. 3777757 3779267.. 4588938 4588938.. 4590449 4921952.. 4923462 6771749.. 6773259 | 32 | 4 | 1141986.. 1143494 2193263.. 2194771 4311768.. 4313276 4852995.. 4854503 |
| 16 | 6 | 113336..114845  595726..597234 1187279.. 1188788 3757209..3758718 4483439..4484948 5083161..5084670 | 33 | 7 | 454542.. 456051 3338359.. 3339868 4611359.. 4612868 4783673.. 4785182 5502220.. 5503729 5507659.. 5509168 6228548.. 6230057 |
| 17 | 5 | 125104.. 126615  851020.. 852531 4740117.. 4741629 5794641.. 5796153 6142030.. 6143542 |  |  |  |
