# Supplementary material for: FN-Identify: Novel Restriction Enzymes-Based Method for Bacterial Identification in Absence of Genome Sequencing
Source: Adv Bioinformatics. 2015 Dec 31;2015:303605. doi: 10.1155/2015/303605 (PMC4735980; doi:10.1155/2015/303605)
Supplement: Supplementary file 1 — The supplementary materials include seven supplementary figures and 12 supplementary tables. Supplementary figure 1 is an illustration of expected restriction results of two Lactobacillus strains. Supplementary figures 2 and 3 are the Identification schemes of Lactobacillus using fragments numbers or fragments numbers and fragments size of HSP60 gene. Supplementary figures 4-7 are the Identification schemes of Pseudomonas and Mycobacterium using fragments numbers only or fragments number and fragments size of 16S RNA gene. Supplementary tables 1-4 list the details of species and strains of Pseudomonas and Mycobacterium that used in this study. Supplementary tables 5-12 are the restriction maps of the species and strains of Lactobacillus, Pseudomonas and Mycobacterium used as input to FN-Identify. [file 303605.f1.zip › Awad-etal-SupplementaryTable3.docx]

**Supplementary table 3: Names and GenBank accession number of Mycobacterium species used in this study**

| **Strain**  **ID*** | **Organism** | **GenBank**  **Accession**  **number** | **Strain**  **ID*** | **Organism** | **GenBank**  **Accession**  **number** |
| --- | --- | --- | --- | --- | --- |
| 1 | *Mycobacterium abscessus* | CU458896 | 12 | *Mycobacterium kansasii* | CP006835 |
| 2 | *Mycobacterium abscessus subsp. bolletii* | CP009447 | 13 | *Mycobacterium leprae* | AL450380 |
| 3 | *Mycobacterium africanum* | FR878060 | 14 | *Mycobacterium liflandii* | CP003899 |
| 4 | *Mycobacterium avium subsp. paratuberculosis* | AE016958 | 15 | *Mycobacterium marinum* | CP000854 |
| 5 | *Mycobacterium bovis subsp. bovis* | BX248333 | 16 | *Mycobacterium neoaurum* | NC_023036 |
| 6 | *Mycobacterium canettii* | HE572590 | 17 | *Mycobacterium rhodesiae* | NC_016604 |
| 7 | *Mycobacterium chubuense* | CP003053 | 18 | *Mycobacterium smegmatis* | CP000480 |
| 8 | *Mycobacterium gilvum Spyr1* | CP002385 | 19 | *Mycobacterium tuberculosis* | AL123456 |
| 9 | *Mycobacterium gilvum PYR-GCK* | CP000656 | 20 | *Mycobacterium ulcerans* | CP000325 |
| 10 | *Mycobacterium indicus* | CP002275 | 21 | *Mycobacterium vanbaalenii* | CP000511 |
| 11 | *Mycobacterium intracellulare* | CP003324 | 22 | *Mycobacterium sp. 05-1390* | CP003347 |
