# Supplementary material for: FN-Identify: Novel Restriction Enzymes-Based Method for Bacterial Identification in Absence of Genome Sequencing
Source: Adv Bioinformatics. 2015 Dec 31;2015:303605. doi: 10.1155/2015/303605 (PMC4735980; doi:10.1155/2015/303605)
Supplement: Supplementary file 1 — The supplementary materials include seven supplementary figures and 12 supplementary tables. Supplementary figure 1 is an illustration of expected restriction results of two Lactobacillus strains. Supplementary figures 2 and 3 are the Identification schemes of Lactobacillus using fragments numbers or fragments numbers and fragments size of HSP60 gene. Supplementary figures 4-7 are the Identification schemes of Pseudomonas and Mycobacterium using fragments numbers only or fragments number and fragments size of 16S RNA gene. Supplementary tables 1-4 list the details of species and strains of Pseudomonas and Mycobacterium that used in this study. Supplementary tables 5-12 are the restriction maps of the species and strains of Lactobacillus, Pseudomonas and Mycobacterium used as input to FN-Identify. [file 303605.f1.zip › Awad-etal-SupplementaryTable4.docx]

**Supplementary table 4: Mycobacterium 16S rRNA copy numbers and positions**

| **Strain**  **ID*** | **16S rRNA**  **Copies**  **number** | **16S rRNA**  **Position** | **Strain**  **ID*** | **16S rRNA**  **Copies**  **Number** | **16S rRNA**  **Position** |
| --- | --- | --- | --- | --- | --- |
| 1 | 1 | 1462397..1463885 | 12 | 1 | 1414940..1416443 |
| 2 | 1 | 3211783..3213291 | 13 | 1 | 1341152..1342669 |
| 3 | 1 | 1474821..1476325 | 14 | 1 | 4639770..4641273 |
| 4 | 1 | 2751283.. 2752788 | 15 | 1 | 5033924..5035427 |
| 5 | 1 | 1469891..1471395 | 16 | 2 | 3588246..3589738  4237596..4239107 |
| 6 | 1 | 1494866..1496370 | 17 | 2 | 3835345..3836837  4695944..4697455 |
| 7 | 2 | 2722536..2724047  3967516..3969008 | 18 | 2 | 3823617..3825111  5027951..5029464 |
| 8 | 2 | 1784693..1786204  2959422..2960914 | 19 | 1 | 1471855..1473359 |
| 9 | 2 | 2414610..2416121  3743082..3744574 | 20 | 1 | 4413404..4414910 |
| 10 | 1 | 1565246..1566749 | 21 | 1 | 3504692..3506186 |
| 11 | 1 | 1392798..1394301 | 22 | 1 | 1405585..1407088 |
